# Pathogenesis of Age-Related Osteoporosis: Impaired Mechano-Responsiveness of Bone Is Not the Culprit

**DOI:** 10.1371/journal.pone.0002540

**Published:** 2008-07-02

**Authors:** Olli V. Leppänen, Harri Sievänen, Jarkko Jokihaara, Ilari Pajamäki, Pekka Kannus, Teppo L. N. Järvinen

**Affiliations:** 1 Medical School and the Institute of Medical Technology, University of Tampere, Tampere, Finland; 2 Division of Orthopaedics and Traumatology, Department of Trauma, Musculoskeletal Surgery and Rehabilitation, Tampere University Hospital, Tampere, Finland; 3 The Bone Research Group, UKK-Institute, Tampere, Finland; 4 Department of Orthopaedics, University of British Columbia, Vancouver, British Columbia, Canada; University of Michigan, United States of America

## Abstract

**Background:**

According to prevailing understanding, skeletal mechano-responsiveness declines with age and this apparent failure of the mechano-sensory feedback system has been attributed to the gradual bone loss with aging (age-related osteoporosis). The objective of this study was to evaluate whether the capacity of senescent skeleton to respond to increased loading is indeed reduced as compared to young mature skeleton.

**Methods and Findings:**

108 male and 101 female rats were randomly assigned into Exercise and Control groups. Exercise groups were subjected to treadmill training either at peak bone mass between 47–61 weeks of age (Mature) or at senescence between 75–102 weeks of age (Senescent). After the training intervention, femoral necks and diaphysis were evaluated with peripheral quantitative computed tomography (pQCT) and mechanical testing; the proximal tibia was assessed with microcomputed tomography (μCT). The μCT analysis revealed that the senescent bone tissue was structurally deteriorated compared to the mature bone tissue, confirming the existence of age-related osteoporosis. As regards the mechano-responsiveness, the used loading resulted in only marginal increases in the bones of the mature animals, while significant exercise-induced increases were observed virtually in all bone traits among the senescent rats.

**Conclusion:**

The bones of senescent rats displayed a clear ability to respond to an exercise regimen that failed to initiate an adaptive response in mature animals. Thus, our observations suggest that the pathogenesis of age-related osteoporosis is not attributable to impaired mechano-responsiveness of aging skeleton. It also seems that strengthening of even senescent bones is possible – naturally provided that safe and efficient training methods can be developed for the oldest old.

## Introduction

**Figure 1:**
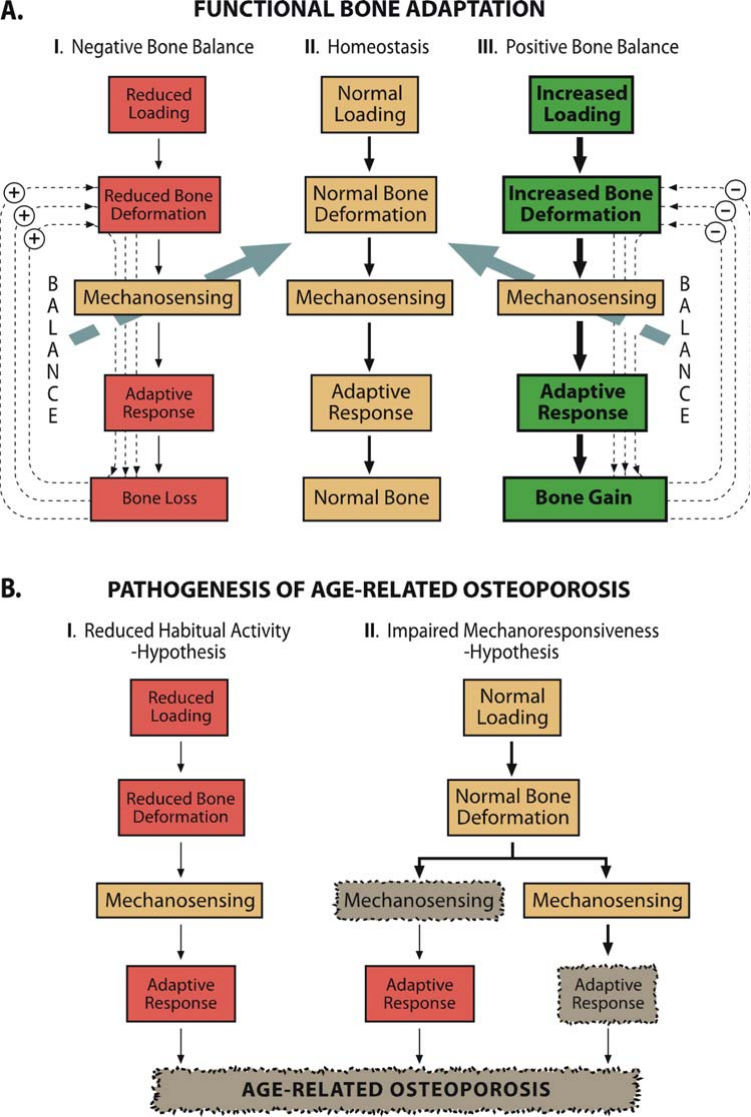
Functional Bone Adaptation (A) and the Proposed Hypothesis for Age-related Osteoporosis (B).

The primary evolutionary function of the bones is to bear the muscle contraction- and gravity-induced mechanical forces exerted on them without breaking, and ultimately, to enable the efficient locomotion of the body [1]. To successfully carry out this locomotive function, the bone tissue is equipped with a mechano-sensory system that facilitates the skeletal adaptation to loading. In essence, bones first *sense* the loading-induced deformation and then elicit a *response* that eventually results in an appropriate modification of the bone structure, if required, to cope with the altered loading milieu (Figure 1A). It has been recently proposed that the pathogenesis of age-related osteoporosis (i.e., the gradual loss of mineral from bones with aging) would be attributable to a failure of this control system [2]: either the mechano-sensitivity of bones is reduced [3], [4] or the capacity of bones to respond to loading is weakened. An alternative pathomechanistic theory suggests that bone loss in senescence represents simply an appropriate response to reduced loading in a less active host [4] (Figure 1B).

Regarding the skeletal mechano-responsiveness *per se*, both systemic factors (hormones such as estrogen and growth hormone) [5]–[12] and local factors (growth factors such as insulin-like growth factor 1 and 2) [13], [14] have been shown to have a direct modulatory effect. Also, individual responses to mechanical stimuli have been shown to depend on genetics [15] and gender [16], [17], whereas the influence of age on bone mechano-responsiveness has remained controversial [3], [18]–[20]. The accumulation of adipocytes to the bone marrow during aging has been speculated to accelerate endocortical resorption [21], whereas it has been shown that periosteal expansion continues well into old age, particularly in men, implying that the mechanosensory system may be properly functioning [22]–[24]. Experimental studies have shown that the responsiveness of the aged skeleton is increased [19], reduced [18], [25], or unaffected [26]–[28]. In our previous study [28], we showed that the ability of bones of young (5–19 week old) and mature (33–47 week old) male rats to adapt to treadmill-running -induced loading was similar, but the adaptive mechanisms differed; in response to given exercise, the growing bones primarily increased cross-sectional size, while the mature bones mainly increased bone density.

Accordingly, the objective of this study was to evaluate whether the skeleton can maintain its capability to respond to increased loading until very old age (senescence). The timing of the increased exercise loading was chosen to coincide appropriate phases of the rat lifespan: maturity and senescence. The mature rats have stopped the longitudinal growth and reached the peak bone mass, while the senescent rats represented the ultimate group in terms of age as judged from more than 50% mortality among control animals at the end of the experiment.

## Materials and Methods

### Animals

The sample size used in this study was based on *a priori* knowledge on natural loss of older animals [29], [30], the expected loss being 20% and 50% in the mature and senescent age groups, respectively, and on the assumed standard deviation of ∼11% in the breaking load of rat femur, the primary outcome [31]. To detect a significant (p<0.05) 10% loading-induced response in the breaking load of femur in the exercised groups (vs. controls) at 80% statistical power, a minimum of ∼15 rats/group was required at the end of the experiment. Accordingly, a total of 108 male rats of the Sprague-Dawley strain were used in the experiment.

**Figure 2:**
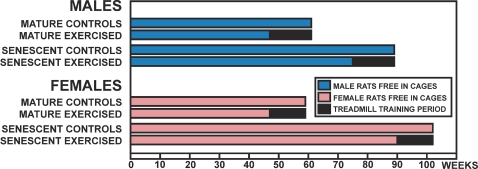
The Design of the Study.

The rats were 3 weeks old at the beginning of the study. During the first 2 weeks of the study, all rats ran on a flat-bed treadmill at a slow speed (10–20 cm/s) for 3 minutes/day for 3 days a week to let the animals to adapt the treadmill running and to exclude those animals refusing to run (about 5% of the original population were removed). The rats were then randomly assigned into four groups: “Mature exercised”, and “Senescent exercised”; and “Mature control”, and “Senescent control” (Table 1, Figure 2). The animals were housed in cages (18×35×55 cm), four animals per cage, at 20°C with a light cycle of 12 h. They were fed standard laboratory chow and water *ad libitum*.

**Table 1:** The Number of Rats at Different Period of the Experiment.

	At the Beginning of the Experiment	At the End of the Experiment
**MALES**		
**MATURE CONTROLS**	23	16
**MATURE EXERCISED**	29	22
**SENESCENT CONTROLS**	24	10
**SENESCENT EXERCISED**	32	14
**FEMALES**		
**MATURE CONTROLS**	22	16
**MATURE EXERCISED**	26	22
**SENESCENT CONTROLS**	23	10
**SENESCENT EXERCISED**	30	17

### Exercise program

Both mature and senescent exercise groups were subjected to a progressive exercise program for 14 weeks (Table 2). The training began at the age of 47 in the Mature exercise group and at 75 weeks Senescent exercise group, respectively (Table 2, Figure 2). To corroborate (or refute) the findings of male rats, a similar experiment was also carried out using 101 female rats. The determination of sample size, as well as the acclimation and randomization procedures were carried out identically to males, but due to the known increased longevity (increased frailty) of the senescent female rats [32], the training protocol and starting age of training were slightly modified in comparison to males (Table 3, Figure 2).

**Table 2:** The Progressive Treadmill Exercise Regimen Used for Male Rats.

Week	Age (weeks)		Duration (min)	Speed (cm/s)	Inclination (deg)
	Mature	Senescent			
1	47	75	5	20	5
2	|	|	10	20	10
3	|	|	10	20	15
4	|	|	10	30	15
5	|	|	10	30	20
6	|	|	10	30	20
7	|	|	10	30	25
8	|	|	10	30	25
9	|	|	10	30	30
10	|	|	10	30	30
11	|	|	10	30	30
12	|	|	10	30	30
13	|	|	10	30	30
14	60	88	10	30	30

**Table 3:** The Progressive Treadmill Exercise Regimen Used for Female Rats.

Week	Age (weeks)		Duration (min)	Speed (cm/s)	Inclination (deg)
	Mature	Senescent			
1	47	90	5	20	5
2	|	|	5	20	10
3	|	|	5	20	15
4	|	|	5	30	15
5	|	|	5	30	20
6	|	|	5	30	20
7	|	|	5	30	25
8	|	|	5	30	25
9	|	|	5	30	30
10	|	|	5	30	30
11	|	|	5	30	30
12	58	101	5	30	30

After the exercise intervention, the exercised animals and their age-matched control animals were euthanized, and body weight and the weight of the uteri, if applicable, were measured. Femora were excised and stored at −20°C in small freezer bags wrapped in saline-soaked gauze bandages to prevent dehydration. This kind of storage has been shown not to affect bone's biomechanical properties [33], [34]. Tibiae were excised and dehydrated in an ethanol series (30 and 70% ethanol) and stored in 70% ethanol. The research protocol was accepted by the Ethics Committee for Animal Experiments of the University of Tampere and the Provincial Government of Western Finland Department of Social Affairs and Health, Finland. The study conformed to the NIH Guide for the Care and Use of Laboratory Animals.

### Bone analysis

At the day of testing, the femora were slowly thawed at the room temperature and kept wrapped in saline-soaked gauzes except during measurements. A digimatic caliper (Mitutoyo 500, Andover, United Kingdom) was used to measure the length of femora. In our laboratory, the coefficient of variation (CV_rms_) for the determination of the length of the femora was 0.2% [35].

### Peripheral quantitative computed tomography

The cross-sections of the femoral diaphysis and neck were scanned with peripheral quantitative computed tomography (pQCT, Stratec XCT Research M, software version 5.40B, Stratec Medizintechnik GmbH, Pforzheim, Germany). For the pQCT assessment of the diaphysis, the femur was inserted into a specially constructed plastic tube with the shaft in axial direction, and one cross-sectional slice was scanned at 50% of the length of the femur [28]. The voxel size was 0.070×0.070×0.5 mm^3^ and the scan speed was 3.0 mm/s. Total cross-sectional area (tCSA), cortical cross-sectional area (cCSA), and cortical bone mineral density (cBMD) were evaluated by the pQCT software using contour mode 1 (threshold 214 mg/cm^3^) for tCSA and separation mode 1 for cCSA and cBMD (threshold 710 mg/cm^3^). In our laboratory, the CV_rms_ in the femoral midshaft were 0.9% for the tCSA, 1.5% for the cCSA, and 0.6% for the cBMD [36].

For the pQCT assessment of the femoral neck, the femur was inserted into a specially constructed plastic tube with the femoral neck in an axial direction [16]. The scan line was adjusted to the midneck using the scout view option of the pQCT software. The voxel size and scan speed were the same as described above. Total cross-sectional area (tCSA), total bone mineral content (tBMC), and total bone mineral density (tBMD) were determined using contour mode 1 (threshold 214 mg/cm^3^) for tCSA, tBMC, and tBMD. In our laboratory, the CV_rms_ were 3.9% for tCSA, 2.2% for tBMC and 2.1% for tBMD [36].

### Mechanical testing

After the pQCT scanning, the right femora were tested mechanically. A Lloyd material testing machine (LR5K, J. J. Lloyd Instruments, Southampton, UK) was used for the anteroposterior three-point bending of the femoral shaft and compression of the femoral neck according to our standard protocols [35], [37].

For the three-point bending, the femur was placed on its posterior surface on the lower supports of the bending apparatus. For each bone, these supports were placed individually (first just distal to the trochanter minor and the other just proximal to the condyles of the femur). After the anatomical adjustment of the supports, a bending load using a brass crossbar was applied to the femoral midshaft perpendicularly to the long axis of the bone until the failure of the specimen. The breaking load (F_max_) of the femoral midshaft was determined from the load-deformation curve. In our laboratory, the CV_rms_ of the F_max_ for three point bending is 5.0% [35].

After the three-point bending of the femoral shaft, the proximal half of femur was mounted in a specially constructed fixation device [38] and a vertical load was applied to the top of the femoral head using a brass crossbar until failure of the femoral neck. The F_max_ of the femoral neck was determined from the load-deformation curve. In our laboratory, the CV_rms_ of the F_max_ for femoral neck compression is 7.6% [39].

### Micro-computed tomography (μCT)

The proximal metaphysis of tibia were scanned using a high resolution micro-computed tomography system (μCT 35; Scanco Medical, Basserdorf, Switzerland) with nominal isotropic resolution of 12 µm. Three-dimensional analysis of trabecular bone was performed on the bone region 1 to 5 mm distal to the growth plate. Trabecular bone compartment was separated from the cortical bone by semi-automatically drawn contours and a global threshold was used to distinguish bone and marrow. The following parameters were determined from the trabecular bone using a direct three-dimensional approach [40]: total bone marrow volume including the trabeculae (TV; mm^3^), trabecular bone volume (BV; mm^3^), trabecular bone volume fraction (BV/TV), mean trabecular number (Tb.N; 1/mm), mean trabecular thickness (Tb.Th; mm), and mean trabecular separation (Tb.Sp; mm). For determination of cortical bone porosity, a 0.5 mm thick region of cortical bone at 7 mm distance from the proximal end of tibia was analyzed.

### Statistical analysis

All data were analyzed using the SPSS for Windows (version 13.0). Relative exercise effects (i.e., the percent difference between exercised and control groups) and aging effects (i.e., the percent difference between mature and senescent groups) were tested using analysis of covariance (ANCOVA), and all data pertaining to mechanical competence of the femur (cCSA, tBMC, tCSA, and F_max_) were statistically controlled for body weight and femoral length [16], [28], [36]. In all tests, an α level less than 5% (p<0.05) was considered statistically significant.

## Results

**Figure 3:**
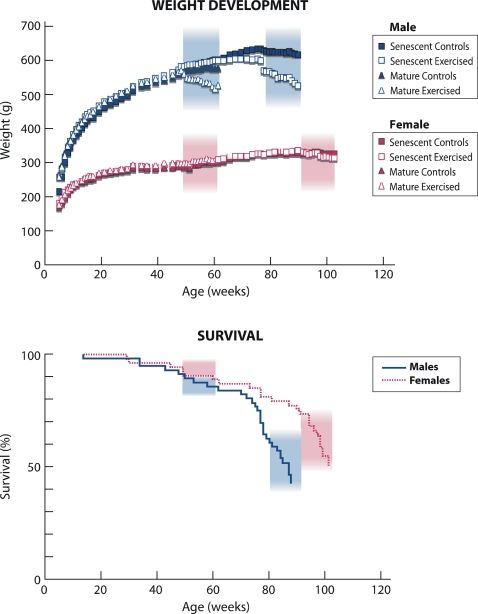
The Body Weight Curves and a Kaplan-Meier Plot Demonstrating the Survival of the Male and Female Rats in This Experiment.

Mortality was 28% and 57% among Mature and Senescent males, respectively (Table 1, Figure 3). The corresponding rates in females were 21% and 49%, respectively (Table 1, Figure 3). Estimated from this mortality, the age of the senescent groups corresponded to over 75 years old men and over 80 years old women in Finland [41]. Figure 3 shows the weight development curves of the rats in each group. The mean weights of the uteri were similar in all female groups.

### Age-related osteoporosis

**Figure 4 pone-0002540-g004:**
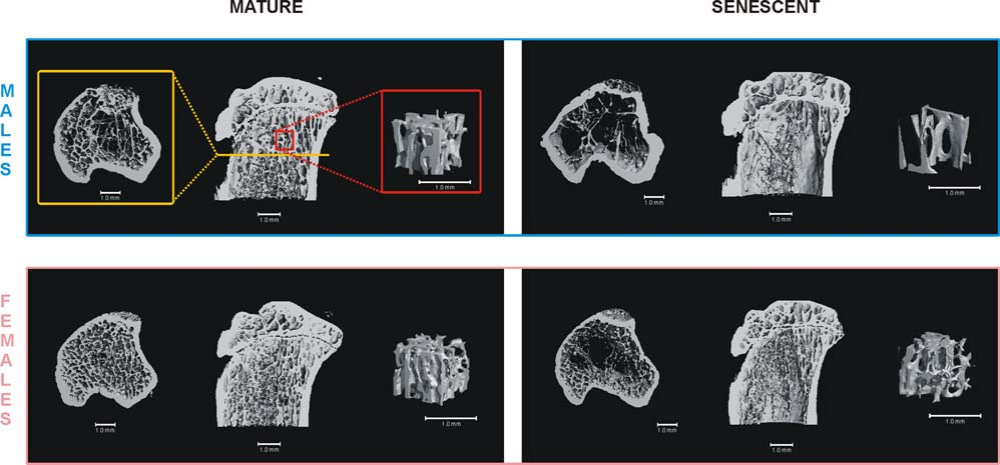
Effects of Aging on the Trabecular Bone Texture in the Proximal Tibial Metaphysis. Due to aging, the proportion of trabecular bone of the bone volume (TV/BV) is decreased in males and females. In addition, in males, the number (Tb.N.) and thickness (Tb.Th.) of the trabeculae is decreased, while the distance between individual trabeculae (Tb.Sp.) is increased.

The influence of aging on bones (Mature vs. Senescent control rats) is summarized in Tables 4 and 5 (grey panels). Senescent control rats had significantly lower F_max_, tBMC and tCSA at the femoral neck in both sexes and F_max_ at the femoral midshaft in females as compared to corresponding Mature control rats. At the femoral midshaft, tCSA of the male rats and cBMD of the female rats were larger in the Senescent groups than in Mature groups. In the proximal tibia, the trabecular bone volume fraction (BV/TV) was significantly decreased in the Senescent rats when compared to the corresponding Mature group both in males and females. In males, also Tb.N., Tb.Sp., and cortical porosity differed significantly between Mature and Senescent groups, a finding in conjunction with reduced BV/TV indicating a deteriorated bone structure among old rats (Figure 4).

**Table 4:** Descriptive Data of the Biomechanical and Tomographic Measurements and Interaction (Difference Between the Two Age-groups in the Exercise-effect) of the Male Rats.

	MATURE		SENESCENT		Age-related change (p)	Mech.responsiveness vs. Age, Interaction (p)
	CONTROL	EXERCISED	CONTROL	EXERCISED		
	MeanSEM	MeanSEM	MeanSEM	MeanSEM		
**BASIC DESCRIPTIVES**						
Body weight (g)	57411	52810^b^	60214	50814^a^	0.076	0.032
Femoral length (mm)	42.00.2	41.90.3	42.00.3	42.30.4	0.334	0.235
**FEMORAL NECK**						
tBMC (mg/mm) *	6.00.1	6.20.1	5.40.2^e^	6.30.2^b^	0.003	0.035
tBMD (mg/cm^3^)	104117	107815	105716	101721^f^	0.470	0.039
tCSA (mm^2^) *	5.90.2	5.80.2	5.20.3^f^	6.10.2^c^	0.024	0.027
Fmax (N) *	1726	1795	1488^f^	1646	0.018	0.647
**FEMORAL MIDSHAFT**						
cBMD (mg/cm^3^)	14815	14746	14729	14617	0.106	0.769
tCSA (mm^2^) *	15.90.3	16.50.3^c^	17.10.4^e^	17.90.4^e^	0.004	0.751
cCSA (mm^2^) *	9.40.2	9.70.2	9.30.3	9.90.2	0.759	0.353
Fmax (N) *	1837	1976	1669	1898	0.130	0.756
**PROXIMAL TIBIA**						
Trabecular TV (mm^3^)	57.11.4	57.73.1	59.13.1	62.11.7	0.509	0.591
Trabecular BV (mm^3^)	8.480.38	8.921.25	7.220.63	6.390.43^f^	0.080	0.319
Trabecular BV/TV (ratio)	0.150.01	0.150.02	0.120.01^f^	0.100.01^e^	0.031	0.227
Tb.N (1/mm)	2.310.06	2.390.13	1.950.10^e^	1.880.06^e^	0.004	0.391
Tb.Th (mm)	0.0850.001	0.0840.003	0.0860.003	0.0800.003	0.725	0.300
Tb.Sp (mm)	0.420.01	0.410.03	0.510.03^e^	0.530.02^e^	0.003	0.512
Cortical porosity (ratio)	0.0070.001	0.0090.001	0.0170.004^e^	0.0160.002^f^	0.007	0.597

^a^ p<0.001, ^b^ p<0.01, ^c^ p<0.05 vs. corresponding control group; ^d^ p<0.001, ^e^ p<0.01, ^f^ p<0.05 vs. corresponding Mature group.

* values adjusted with body weight and femoral length; for details, see Statistical analysis.

tBMC, total bone mineral content; tBMD, total bone mineral density; tCSA, total cross-sectional area; Fmax, breaking load; cBMD, cortical bone mineral density; cCSA, cortical cross-sectional area; TV, total bone marrow volume; BV, bone volume; Tb.N, mean trabecular number; Tb.Th, mean trabecular thickness; Tb.Sp, mean trabecular separation.

**Table 5:** Descriptive Data of the Biomechanical and Tomographic Measurements and Interaction (Difference Between the Two Age-groups in the Exercise-effect) of the Female Rats.

	MATURE		SENESCENT		Age-related change (p)	Mech.responsiveness vs. Age, Interaction (p)
	CONTROL	EXERCISED	CONTROL	EXERCISED		
	MeanSEM	MeanSEM	MeanSEM	MeanSEM		
**BASIC DESCRIPTIVES**						
Body weight (g)	3079	3127	3137	2985	0.634	0.208
Femoral length (mm)	35.90.2	36.60.2^c^	36.00.3	36.00.2	0.901	0.173
Uterus weight (g)	1.50.1	1.40.1	1.90.2	1.50.1	0.123	0.158
**FEMORAL NECK**						
tBMC (mg/mm) *	5.10.1	5.00.1	4.70.1^f^	5.20.1^b^	0.024	0.002
tBMD (mg/cm^3^)	112915	11938^a^	116620	116414	0.155	0.022
tCSA (mm^2^) *	4.50.1	4.20.1^c^	4.00.1^f^	4.50.1^cf^	0.015	0.001
Fmax (N) *	1245	1304	1016^e^	1195^c^	0.008	0.226
**FEMORAL MIDSHAFT**						
cBMD (mg/cm^3^)	14862	14882	14974^e^	14994^f^	0.009	0.933
tCSA (mm^2^) *	10.70.1	10.70.1	10.80.2	11.10.1^f^	0.648	0.247
cCSA (mm^2^) *	6.60.1	6.50.1	6.50.1	6.80.1^f^	0.424	0.055
Fmax (N) *	1444	1464	1235^f^	1444^c^	0.014	0.032
**PROXIMAL TIBIA**						
Trabecular TV (mm^3^)	38.72.0	38.62.4	39.31.4	38.51.2	0.820	0.851
Trabecular BV (mm^3^)	9.601.02	9.950.80	7.280.76	7.790.42^f^	0.087	0.904
Trabecular BV/TV (ratio)	0.250.02	0.260.01	0.190.02^f^	0.200.01^e^	0.048	0.666
Tb.N (1/mm)	3.560.29	3.840.14	2.990.15	3.140.10^d^	0.108	0.694
Tb.Th (mm)	0.0770.004	0.0730.001	0.0700.003	0.0730.002	0.204	0.187
Tb.Sp (mm)	0.280.04	0.240.01	0.320.02	0.300.01^d^	0.283	0.704
Cortical porosity (ratio)	0.0060.000	0.0070.001	0.0070.001	0.0060.000	0.205	0.058

^a^ p<0.001, ^b^ p<0.01, ^c^ p<0.05 vs. corresponding control group; ^d^ p<0.001, ^e^ p<0.01, ^f^ p<0.05 vs. corresponding Mature group.

* values adjusted with body weight and femoral length; for details, see Statistical analysis.

tBMC, total bone mineral content; tBMD, total bone mineral density; tCSA, total cross-sectional area; Fmax, breaking load; cBMD, cortical bone mineral density; cCSA, cortical cross-sectional area; TV, total bone marrow volume; BV, bone volume; Tb.N, mean trabecular number; Tb.Th, mean trabecular thickness; Tb.Sp, mean trabecular separation.

### Exercise effects

#### Body weight and femoral length

In males, there was a significant exercise-related decrease in body weight: −8.2% (p = 0.005) and −15.7% (p<0.001) in Mature and Senescent groups, respectively (Table 4). In females, body weight was not influenced by exercise (Table 5). Femoral length was similar between exercised and control rats in male groups; whereas in Mature females the femur was 1.7% longer in exercise group than in control group (p = 0.043).

#### The geometric, densitometric, and biomechanical bone traits

**Figure 5:**
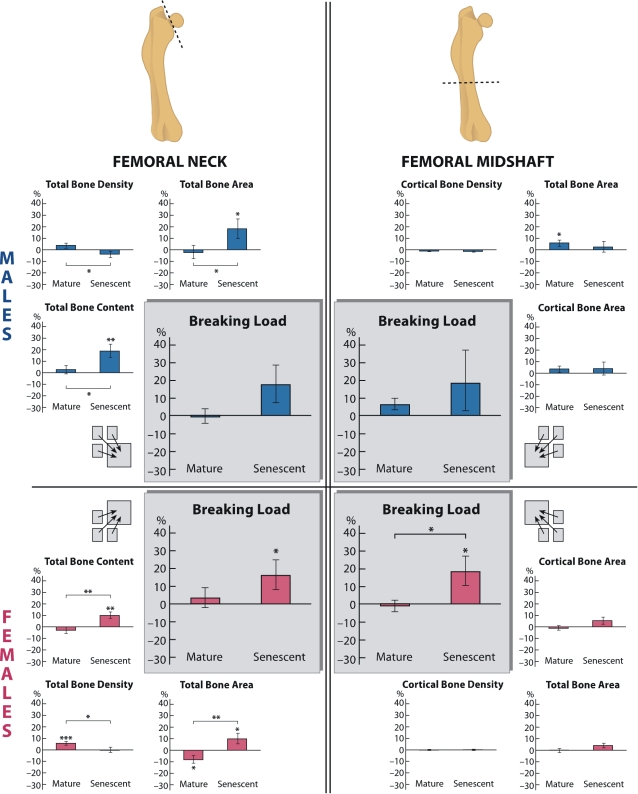
Exercise Effect on Different Bone Traits of the Femoral Neck and Femoral Midshaft in Mature and Senescent Male and Female Rats. Bars represent percent (%) increases (± the standard error of the mean, SEM) of the exercise group compared to corresponding control group at the end of the treadmill exercise intervention in the femoral neck total bone content (tBMC); total bone density (tBMD); total bone area (tCSA); cortical bone density (cBMD); cortical bone area (cCSA); and breaking load (F_max_). Significant differences between the exercised rats and their controls, and between the two age-groups in the exercise-effect, are indicated: *p<0.05; **p<0.01; ***p<0.001. Results for tBMC, tCSA, cCSA, and F_max_ are adjusted for body weight and femoral length.

Skeletal responses to increased exercise among the Mature and Senescent rats are depicted in Tables 4 and 5 and Figure 5. In the Mature groups, significant exercise-induced increases were observed: total cross-sectional area (tCSA) at the femoral diaphysis of the males increased 6% (p = 0.018) compared to age-matched controls, and total bone mineral density (tBMD) at the femoral neck of the females increased 6% (p<0.001) while its tCSA remained 8% (p = 0.018) smaller compared to controls. Among the senescent rats significant exercise-induced between-group effects were observed virtually in all bone traits; both tCSA and bone mineral content (tBMC) at the femoral neck increased 19% (p = 0.003) and 18% (p = 0.030) in males and 10% (p = 0.026) and 10% (p = 0.001) in females, respectively. Also, breaking load (F_max_) both at the femoral neck and femoral diaphysis of senescent females increased 16% (p = 0.045) and 19% (p = 0.026), respectively; while in the senescent males F_max_ at the femoral neck increased 18% (p = 0.087). No differences between exercised and control rats were observed in proximal tibia in any of the bone traits determined using micro-CT analysis.

### Age and the mechano-responsiveness of bone

An age-effect on bone mechano-responsiveness (interaction between age and exercise loading) was observed at the femoral neck. The exercise-effect was significantly greater in the Senescent group for tBMC (p = 0.035 and p = 0.002) and tCSA (p = 0.027 and p = 0.001) both in males and females, respectively (Tables 4 and 5 and Figure 5). The accompanying significant decrease in tBMD (p = 0.039 and p = 0.022, in males and females respectively) indicated that the exercise-effect was more pronounced in tCSA than in tBMC. As regards bone strength, the mean exercise-effects on F_max_ were greater in the Senescent group, but the group-difference reached statistical significance only at the femoral diaphysis in females (p = 0.032) (Figure 5).

## Discussion

Bone functional bone adaptation [42]–[46] is one of the cardinal principles in skeletal biology depicting a homeostatic feedback system evolved to maintain the skeletal integrity in different loading milieus through appropriate modifications in bone geometry and structure, and/or material properties - with or without changes in bone mass. Accordingly, any substantial change either in the sensitivity of the mechano-sensory system or in the balance between predominant bone loading and coexisting bone rigidity results in an adaptive response to keep the tissue deformations within the predetermined physiological window [42], [43], [45]. In this context, the occurrence of age-related osteoporosis, or the gradual bone loss with aging, has been attributed to the failure of this mechano-sensory mechanism [3], [4]. In our experiment, the senescent rats displayed a clear age-related osteoporosis, manifest as deteriorated bone structure and reduced bone structural strength (Tables 4 and 5). Nevertheless, these animals also showed a positive adaptive response to exercise while much less consistent response was seen in the mature rats subjected to the same exercise regimen (Figure 5). This finding challenges the reduced mechano-sensitivity at senescence as the pathomechanism of age-related osteoporosis.

We therefore speculate that the enhanced mechano-responsiveness among the senescent animals was attributable to the apparent fact that their bones were initially less rigid because of essentially diminished habitual activity in aged rats [47]. However, as a consequence of additional treadmill training, the bones were subjected to increased loading, that being clearly beyond that experienced during normal living in terms of magnitude and intensity. These exercise-induced deformations then resulted in the adaptive response observed in the bones of Senescent animals. In the Mature rats, in turn, their fully developed skeleton and relatively higher habitual activity ensured readily an appropriate mechanical competence for the treadmill running, and there remained only a marginal room to respond to mechanical stimulus caused by additional treadmill training. These observations also suggest a biomechanical explanation for the apparent *direct* modulatory effect of aging on the periosteal apposition: rather than originating from the effect of aging *per se* on the periosteum, it seems that the aging-associated periosteal enlargement is an adaptive response to cope with endocortical loss of mineral (the imminent decrease in bone rigidity). As described above, any change either in the loading subjected on the bone or its strength (structural rigidity) necessitates an adaptive response to restore the delicately controlled stress-strain equilibrium.

Although our study was a randomized controlled trial using rats of controlled genetics, large sample size, long intervention period and well-validated methodology [16], [28], [36], [48], [49], it had some limitations that require consideration. First, bone deformations during running were not measured. Instead, our conclusions relied on a simple engineering principle that equal loading imposed on a less rigid bone produces greater deformations and consequently larger response and vice versa. Thus, it needs to be noted that our paper does not deal with the mechano-sensitivity of bones between Mature and Senescent animals. As discussed above, the treadmill training -induced strain stimulus may not have been sufficient for bone formation activity [50], [51] for mature animals with inherently more rigid bones, while a more vigorous loading would have been necessary to induce an osteogenic response in mature animals. Here the quite liberally used terms ‘mechano-sensitivity’ and ‘mechano-responsiveness’ need to be distinguished from each other. In the most stringent sense, these two terms depict distinct phases of functional bone adaptation -cascade (Figure 1A). It is indeed possible that aging disproportionately affects the skeletal mechano-sensing and responsiveness (Figure 1B) and a failure in the former could be only verified with direct strain measurements; i.e., a similar strain environment would lead to smaller response among old animals than among younger, mature animals. However, notwithstanding this possibility, we highlight that our finding of a significant *adaptive response* to increased exercise loading (i.e., increase in most bone traits, including bone strength) in senescent animals shows that the homeostatic control system of the skeleton functions even in the very old age and the skeletal responsiveness is not impaired.

One might find the lack of exercise-induced increases in bone characteristics in the mature animals somewhat controversial to findings of our previous study [28], in which the exercise-induced benefits were seen among adult male rats (33 to 47-week-old during the study) subjected to the same treadmill training protocol. However, in that study, the adult animals were still growing axially. We therefore feel that the observed difference in the skeletal responsiveness between these two groups of mature animals actually underpins the importance of the longitudinal growth period as an opportune window to enhance of impact of mechanical loading on bone [52]–[60]. Also, the present senescent rats represent the extreme in terms of age; in agreement with the increased mortality, the aged animals displayed deteriorated bone traits and a decreased body weight (particularly in males) (Figures 3–5 and Tables 4 and 5), all changes characteristic of senescence [61].

The present findings do not allow one to make conclusions about the potential influence of gender on the mechano-responsiveness of bones, since there were apparent differences in the survival and functional capacity of the aged animals rendering the study designs in males and females basically different (distinct age at entry of the initiation of exercise in senescent animals and different treadmill training protocols). In essence, due to the increased longevity of female rats and the resulting increased frailty, we felt compelled to subject the senescent females to a less physically challenging exercise regimen. However, the effect of gender on the skeletal responsiveness to loading has been previously assessed [16], [17], [62]–[66], suggesting that males are more responsive to loading than females.

In conclusion, our results demonstrate that concerning the mass, structure, and mechanical competence of rat bones, the homeostatic loading-driven regulatory feedback system maintains its capacity to respond to increased exercise loading even into very old age. Accordingly, it is unlikely that the pathogenesis of age-related osteoporosis would be attributable solely, if at all, to a failure in this system. Thus, our observations suggest that strengthening of senescent human bones is also possible – naturally provided that safe and efficient training methods can be developed for the oldest old.
