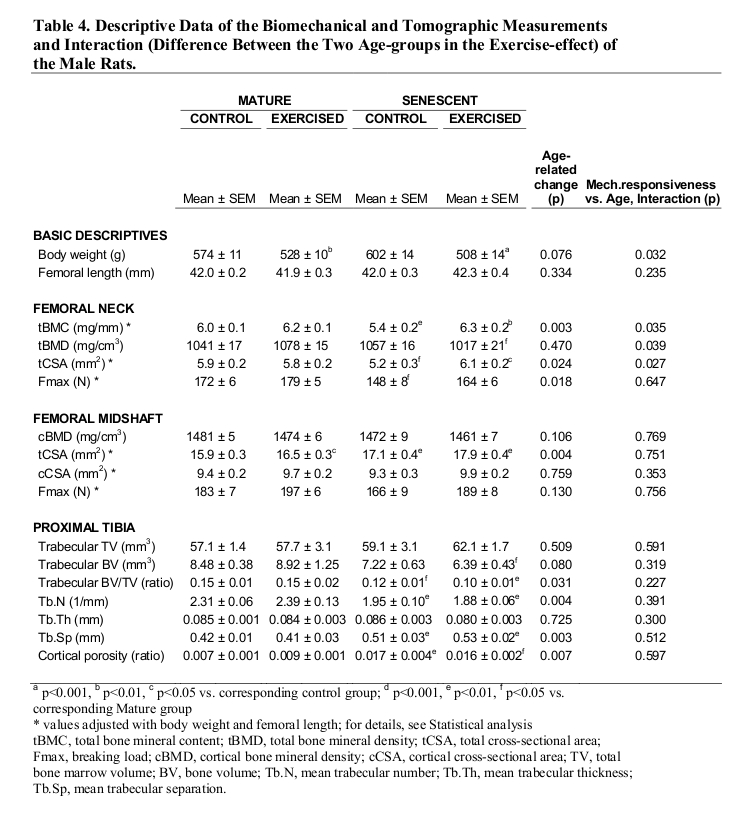# Correction: Pathogenesis of Age-Related Osteoporosis: Impaired Mechano-Responsiveness of Bone Is Not the Culprit

**DOI:** 10.1371/annotation/6083a0e9-04e7-46dc-bb6d-9de65579d2bb

**Published:** 2008-08-20

**Authors:** Olli V. Leppänen, Harri Sievänen, Jarkko Jokihaara, Ilari Pajamäki, Pekka Kannus, Teppo L. N. Järvinen

The plus/minus symbols were left out of the second through fifth columns of Table 4. Please view the correct Table 4 here:

**Figure:**