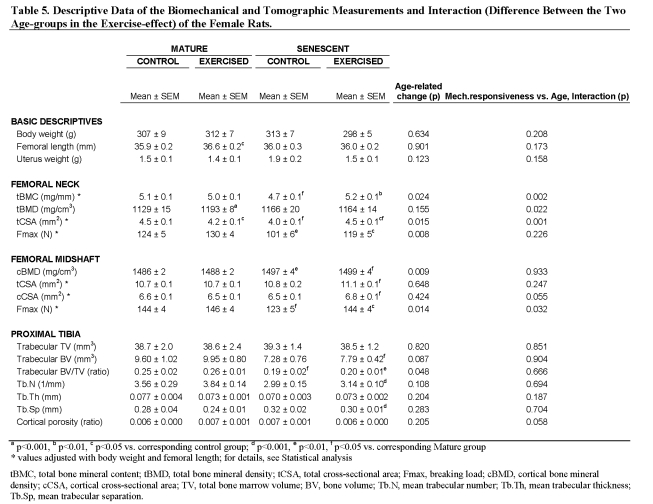# Correction: Pathogenesis of Age-Related Osteoporosis: Impaired Mechano-Responsiveness of Bone Is Not the Culprit

**DOI:** 10.1371/annotation/5d8eaa9e-b4b2-4b90-8fa6-242ba94fac5e

**Published:** 2008-08-20

**Authors:** Olli V. Leppänen, Harri Sievänen, Jarkko Jokihaara, Ilari Pajamäki, Pekka Kannus, Teppo L. N. Järvinen

The plus/minus symbols were left out of the second through fifth columns of Table 5. Please view the correct Table 5 here:

**Figure:**